# Barriers to Optimal Clinician Guideline Adherence in Management of Markedly Elevated Blood Pressure: A Qualitative Study

**DOI:** 10.1001/jamanetworkopen.2024.26135

**Published:** 2024-08-06

**Authors:** Yuan Lu, Oreoluwa Arowojolu, Xiaoliang Qiu, Yuntian Liu, Leslie A. Curry, Harlan M. Krumholz

**Affiliations:** 1 Center for Outcomes Research and Evaluation, Yale New Haven Hospital, New Haven, Connecticut; 2 Section of Cardiovascular Medicine, Department of Internal Medicine, Yale School of Medicine, New Haven, Connecticut; 3 Department of Internal Medicine, Yale School of Medicine, New Haven, Connecticut; 4 Department of Health Policy and Management, Yale School of Public Health, New Haven, Connecticut

## Abstract

**IMPORTANCE:**

Hypertension poses a substantial public health challenge. Despite clinical practice guidelines for hypertension management, clinician adherence to these guidelines remains suboptimal.

**OBJECTIVE:**

To develop a taxonomy of suboptimal adherence scenarios for severe hypertension and identify barriers to guideline adherence.

**DESIGN, SETTING, and PARTICIPANTS:**

This qualitative content analysis using electronic health records (EHRs) of Yale New Haven Health System included participants who had at least 2 consecutive visits with markedly elevated blood pressure (BP; defined as at least 2 consecutive readings of systolic BP ≥160 mm Hg and diastolic BP ≥100 mm Hg) between January 1, 2013, and December 31, 2021, and no prescription for antihypertensive medication within a 90 days of the second BP measurement. Data analysis was conducted from January to December 2023.

**MAIN OUTCOMES AND MEASURES:**

The primary outcome was scenarios and influencing factors contributing to clinician nonadherence to the guidelines for hypertension management. A thematic analysis of EHR data was conducted to generate a pragmatic taxonomy of scenarios of suboptimal clinician guideline adherence in the management of severe hypertension.

**RESULTS:**

Of the 20 654 patients who met criteria, 200 were randomly selected and thematic saturation was reached after analyzing 100 patients (mean [SD] age at index visit, 66.5 [12.8] years; 50 female [50%]; 8 Black [8%]; 5 Hispanic or Latino [5%]; 85 White [85%]). Three content domains emerged: (1) clinician-related scenarios (defined as noninitiation or nonintensification of treatment due to issues relating to clinician intention, capability, or scope), which included 2 subcategories (did not address and diffusion of responsibility); (2) patient-related scenarios (defined as noninitiation or nonintensification of treatment due to patient behavioral considerations), which included 2 subcategories (patient nonadherence and patient preference); and (3) clinical complexity–related scenarios (defined as noninitiation or nonintensification of treatment due to clinical situational complexities), which included 3 subcategories (diagnostic uncertainty, maintenance of current intervention, and competing medical priorities).

**CONCLUSIONS AND RELEVANCE:**

In this qualitative study of EHR data, a taxonomy of suboptimal adherence scenarios for severe hypertension was developed and barriers to guideline adherence were identified. This pragmatic taxonomy lays the foundation for developing targeted interventions to improve clinician adherence to guidelines and patient outcomes.

## Key Points

**Question:**

What are the plausible scenarios and factors contributing to clinician nonadherence to the guidelines for hypertension management?

**Findings:**

In this qualitative study of 100 patients with markedly elevated blood pressure, 3 domains of suboptimal adherence were developed (clinician-related scenarios, patient-related scenarios, and clinical complexity–related scenarios) and several plausible contributing factors were identified, including a lack of clear protocols and processes to implement guidelines, infrastructure limitations, clinicians’ lack of autonomy and authority, excessive workload, time constraints, and clinician belief or perception.

**Meaning:**

This study introduced a taxonomy poised to inform targeted interventions, thereby enhancing guideline adherence and elevating care quality for severe hypertension.

## Teaser

This qualitative analysis uses electronic health record data to analyze barriers to clinician adherence to clinical guidelines for managing severe hypertension.

## Introduction

Hypertension is a major public health issue, affecting almost one-half of the US adult population. Among patients with hypertension, those with severely elevated blood pressure (BP; defined as at least 2 consecutive readings of systolic BP ≥160 mm Hg or diastolic BP ≥100 mm Hg) comprise about 12%^1,2^ and face a higher risk of complications, including severe and rapid systemic end-organ damage, compared with those with modestly elevated BP.^3^ This condition requires prompt and appropriate pharmacological treatment. The 2017 American College of Cardiology and American Heart Association guidelines^3^ recommend immediate evaluation and drug treatment followed by careful monitoring and dose adjustments for patients with severe hypertension. Despite these well-established clinical practice guidelines, clinician adherence remains suboptimal. A recent study^4^ based on electronic health record (EHR) data in the ambulatory setting found that almost 30% of patients with severely elevated BP had no active antihypertensive drug prescription before their second visit, and only 54% of those who were prescribed at least 1 antihypertensive drug class were prescribed the guideline-recommended 2-drug class combination therapy. This finding highlights a missed opportunity to improve guideline adherence in this population.

Clinicians’ adherence to medication guidelines is a complex and multifaceted process that substantially impacts the implementation of evidence-based practice.^5^ The literature highlights various scenarios resulting in nonadherence to medication guidelines. These scenarios include situations where the recorded BP does not accurately reflect the patient’s typical BP, such as when home BPs are below the target range or when the patient is experiencing pain.^6^ In addition, scenarios such as the prioritization of other clinical concerns over hypertension, the need for ongoing monitoring and lifestyle counseling, and disagreements with specific recommendations, also result in nonadherence.^6^ Moreover, how clinicians address patient-level factors, such as medication nonadherence and individual patient preferences, substantially influences guideline adherence. Clinician-level factors, including the belief that hypertension management is another clinician’s responsibility, further impact guideline adherence. Medication-related issues, such as adverse drug events and use of medications from external sources, present additional adherence challenges.^6^ By recognizing and addressing these multifaceted factors, health care systems can implement strategies to improve clinician adherence to medication guidelines and enhance patient outcomes.

However, the current body of research on clinician guideline adherence in managing markedly elevated BP lacks a comprehensive identification of the reasons behind the inadequate treatment, particularly those based on routinely collected information during clinical practice, such as data from medical records. This information is particularly crucial as pharmacological interventions are vital in reducing BP and associated complications for this patient population. Furthermore, previous studies may have inadequately reported or underrepresented barriers to clinician guideline adherence, potentially due to methodological limitations.^5^ Consequently, we aimed to address these gaps by conducting a content analysis of EHRs to develop a comprehensive taxonomy of scenarios representing suboptimal guideline adherence in the ambulatory management of severe hypertension. This information can potentially guide the creation and implementation of focused interventions, enhancing adherence to guidelines and quality of care for severe hypertension. Moreover, because the information is derived from EHR data, it is pragmatic and enables the development of practical, automated EHR-based clinical decision support tools.^7^

## Methods

### Data Sources

This qualitative study was approved by the institutional review board at Yale University and the need for informed consent was waived. This study was reported according to the Standards for Reporting Qualitative Research (SRQR) reporting guideline.^8^ The dataset included data from adult patients at Yale New Haven Health System (YNHHS) who had at least 2 consecutive outpatient visits between January 1, 2013, and December 31, 2021. YNHHS is a large academic health system comprising 5 distinct hospitals and their associated ambulatory clinics in Connecticut and Rhode Island. All YNHHS hospitals used a secure, centralized EHR system designed by Epic Corporation to collect and store clinical and administrative data. The EHR data are maintained in a data repository at the YNHHS server.

### Study Population

Eligible patients were aged 18 to 85 years and had markedly elevated BP, defined as having measurements of systolic BP of 160 mm Hg or greater or diastolic BP of 100 mm Hg or greater in at least 2 consecutive outpatient visits between January 1, 2013, and December 31, 2021, with no new antihypertensive medication prescription within 90 days of the index date. The index date was defined as the date of the second severely elevated BP reading. Patients with markedly elevated BP were selected as a focus given that the need to urgently achieve BP control in this population is unequivocal. Any 2 consecutive visits were required to be at least 1 day apart. We had access to all available data in the medical records, including patient demographics, past medical histories, vital signs, outpatient medications, laboratory results, encounter notes and scanned documents. Of note, identification of patient race and ethnicity was conducted using data extracted from the EHR. This data was classified based on information provided directly by the patients themselves, either through self-report at the time of registration or during patient intake processes. The specific categories for race included in our study were Black, White, and other (defined as any race not otherwise specified), while ethnicity is categorized as Hispanic or Latino, non-Hispanic, and other (defined as any ethnicity not otherwise specified). A total of 20 654 patients met the eligibility criteria (eFigure in Supplement 1). We randomly selected 200 records from the group of all eligible patients for qualitative analysis, intending to select more if we did not achieve saturation (where no new concepts emerged from analyses of subsequent data^9^).

### Approach to Thematic Analysis and Taxonomy Development

Using a previously published inductive, systematic approach,^10,11,12,13,14^ we conducted a thematic analysis of EHR data to generate a pragmatic taxonomy of suboptimal clinician guideline adherence scenarios in managing severe hypertension.

#### Step 1: Development of Rubric for Medical Record Review

Through an iterative process, a team of 3 clinicians and/or experienced cardiovascular researchers (O.A., Y.L., and H.M.K.) developed a rubric to systematically abstract data from the EHR. We obtained demographic data (including age, sex, race, and ethnicity) and clinical data relevant to the diagnosis and treatment of hypertension (including BP measurements, medical history, medication prescriptions, and medical context of the encounter) and established criteria for consistency (to support explicit review). Additionally, the data extraction rubric was designed to offer flexibility, allowing reviewers to go beyond strict numerical or binary criteria and make subjective assessments. This approach included evaluating the rationale behind a clinician’s decisions, considering the medical context of each encounter, and interpreting data points with a nuanced understanding of patient history, comorbidities, or unique clinical scenarios. Furthermore, while the rubric establishes consistency criteria, it also provides guidance for implicit review, enabling reviewers to use their clinical judgment to uncover underlying reasons for suboptimal adherence to guidelines not explicitly stated in the EHR (implicit review).^14,15,16,17^

#### Step 2: Data Abstraction

Two abstractors (O.A. and X.Q.) participated in a training session, during which they collectively abstracted 15 medical records using the rubric and generated a narrative summary for each case. Decision rules and operational definitions were refined to reduce ambiguity and to facilitate standardized data abstraction. Discrepancies were resolved during face-to-face meetings with discussion among all reviewers until consensus was reached. Once the rubric was finalized, each abstractor reviewed a random sample of 50 medical records. Cases were reviewed until reviewers determined they reached saturation; that is, no new constructs emerged from reviewing subsequent cases.^9^ Specifically, when reviewers felt they reached saturation, they reviewed another 10 charts to confirm no further constructs were identified.

#### Step 3: Content Analysis and Taxonomy Development

The cases abstracted using the rubric were analyzed using conventional content analysis. Content analysis is a systematic, replicable technique for compressing many words of text into fewer content categories based on explicit coding rules.^18,19^ Content analysis enables researchers to sift through large volumes of data with relative ease in a systematic fashion, and it is useful in examining the patterns in documentation.^20^

We used emergent coding and established categories following a preliminary examination of the abstracted data obtained in step 2. First, 1 author (O.A.) independently reviewed the abstracted data and identified a set of suboptimal clinician guideline adherence scenarios to form the initial code list, which was then developed into a consolidated code book. Second, 2 authors (O.A. and Y.L.) reviewed this code book for face validity and revised it based on group discussion. Third, the consolidated code book was trialed on 10 cases by the coding group (O.A. and Y.L.) to ensure consistent coding application. The coding group checked that the reliability of the coding was established (agreement >95%). Then all cases were coded by the coding group. Finally, a larger author group (O.A., Y.L., L.C., and H.M.K.) used an iterative, consensus-based discussion process to group the coding into major content themes with subthemes, maintaining a consensus and primary data referencing approach.^21^

### Data Analysis

The thematic analysis and qualitative data were analyzed using NVIVO software version 12.0 (QSR International). The analysis was conducted from January to December 2023.

## Results

### Study Sample Characteristics

Thematic saturation was reached after analyzing 100 patients. These 100 patients (mean [SD] age at index visit, 66.5 [12.8] years; 50 female [50%]; 8 Black [8%]; 5 Hispanic or Latino [5%]; 85 White [85%]) were included in the final content analysis (Table 1). A total of 31 patients (31.0%) had private insurance, 58 (58%) had Medicare, and 11 (11%) had Medicaid; there were no participants without health insurance. The mean (SD) systolic BP and diastolic BP of the sample at the index date was 166.2 (11.5) mmHg and 87.7 (12.7) mmHg, respectively. The median (IQR) time between visits was 42 (18-85) days. A large proportion of patients had comorbidities at the index date, including 23 patients (23%) with obesity (body mass index ≥30 [calculated as weight in kilograms divided by height in meters squared]), 16 (16%) with diabetes, 31 (31%) with dyslipidemia, and 36 (36%) with cancer.

**Table 1.** Demographics of 100 Patients Included in the Sample for Qualitative Content Analysis

Characteristics	Patients, No. (%) (N = 100)
Age, mean (SD), y	66.5 (12.8)
Sex	
Female	50 (50)
Male	50 (50)
Race	
Black	8 (8)
White	85 (85)
Other or unknown^a^	7 (7)
Ethnicity	
Hispanic or Latino	5 (5)
Non-Hispanic	92 (92)
Other or unknown^a^	3 (3)
Insurance type	
Medicaid	11 (11)
Medicare	58 (58)
Private	31 (31)
None	0
Body mass index category^b^	
≥30	23 (23)
25 to <30	29 (29)
<25	41 (41)
Unknown	7 (7)
Comorbidities	
Heart failure	1 (1)
Diabetes	16 (16)
Dyslipidemia	31 (31)
Coronary artery disease	8 (8)
Chronic kidney disease	5 (5)
Chronic obstructive pulmonary disease	4 (4)
Cancer	36 (36)
Peripheral arterial disease	4 (4)
Depression	6 (6)
Systolic blood pressure at the index date, mean (SD), mm Hg	166.2 (11.5)
Diastolic blood pressure at the index date, mean (SD), mm Hg	87.7 (12.7)
Site of care at the index date	
Primary care, internal medicine, or family medicine	18 (18)
Cardiology	3 (3)
Endocrinology	4 (4)
Oncology	25 (25)
Surgery	11 (11)
Urology	10 (10)
Other	29 (29)

^a^ Other race and ethnicity were defined as any race or ethnicity not otherwise specified.

^b^ Body mass index was calculated as weight in kilograms divided by height in meters squared.

### Content Domains

Based on a thematic analysis of data available in the EHR for patients meeting our criteria, we identified a variety of scenarios of suboptimal clinician guideline adherence in managing severe hypertension pertaining to either noninitiation or nonintensification of pharmacological therapy (Table 2). Noninitiation of pharmacological treatment was defined as an absence of the initiation of antihypertensive therapy in response to severely elevated BP in a patient with at least 2 consecutive readings of severely elevated BP. Nonintensification of pharmacological treatment was defined as failure to intensify or modify treatment or initiate an urgent referral on the index visit for a patient with severely elevated BP who was previously taking antihypertensive medication.

**Table 2.** Scenarios of Suboptimal Clinician Guideline Adherence in the Management of Severe Hypertension Identified Under Noninitiation or Nonintensification of Antihypertensive Treatment (Order of Frequency From Most to Least)

Suboptimal treatment type	Scenario
Noninitiation of treatment	Did not address
	Patient preference
	Diffusion of responsibility
	Diagnostic uncertainty with blood pressure measurement
Nonintensification of treatment	Did not address
	Diffusion of responsibility
	Patient nonadherence
	Patient preference
	Diagnostic uncertainty with blood pressure measurement
	Maintenance of current blood pressure intervention
	Competing medical priorities

These identified scenarios (subcategories) of suboptimal clinician guideline adherence were taxonomized and grouped into 3 main content domains: clinician-related scenarios, patient-related scenarios, and clinical complexity–related scenarios (Figure). Table 3 includes example quotations or clinical situations pertaining to each scenario.

**Figure.**
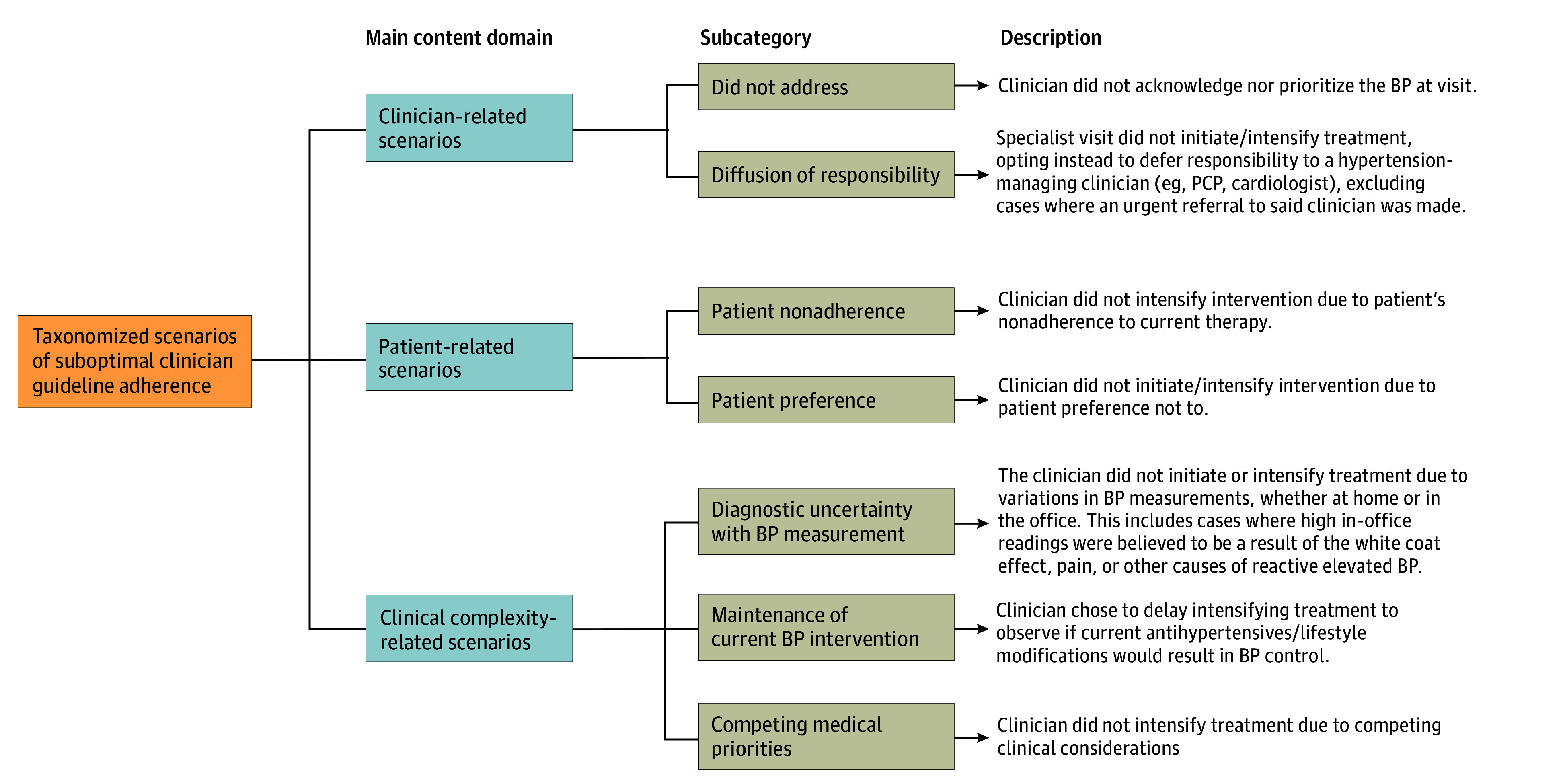
Taxonomy of Scenarios of Suboptimal Clinician Guideline Adherence in the Management of Severe Hypertension BP indicates blood pressure; PCP, primary care physician.

**Table 3.** Example Quotations and Clinical Situations Illustrating Clinician-Related, Patient-Related, and Clinical Complexity–Related Scenarios

Main content domain and subcategory	Example quotation or clinical situation
Clinician-related scenarios	
Did not address	Patient came in for wound care. BP was not addressed in encounter note.
Diffusion of responsibility	“Blood pressure is elevated. Patients needs to see his primary MD for hypertension.” – Podiatrist
Patient-related scenarios	
Patient nonadherence	“Patient noted to take metoprolol, however he has not taken his medications for two days…this would explain his tachycardia and increase in NIBP measurement.”
Patient preference	“Patient states that he has some element of white coat hypertension and that his blood pressure tends to run lower. Does not want medication at this time.”
Clinical complexity–related scenarios	
Diagnostic uncertainty with BP measurement	“BP on arrival 170/98 mmHg. Patient states she was advised to follow up with PCP after appointments at cancer center in February. Patient stated she did, but that her blood pressure is only elevated when she is at cancer center.”
Maintenance of current BP intervention	“Continue meds. Continue BP check at home.”
Competing medical priorities	“We would recommend no treatment at this time…especially since she has significant history of kidney injury.”

Abbreviations: BP, blood pressure; MD, medical doctor; NIBP, noninvasive blood pressure; PCP, primary care physician.

#### Clinician-Related Scenarios

Clinician-related scenarios were defined as instances where clinicians did not initiate or intensify antihypertensive treatment due to factors related to their intentions, capabilities, or scope. Under this main content domain, we identified 2 subcategories: did not address and diffusion of responsibility (Figure). Did not address included instances in which the clinician encountered on the index date neither acknowledged nor prioritized the BP at the visit. For example, Table 3 highlights a clinical scenario in which a patient who presented to a clinician for wound care had a second consecutive markedly elevated BP reading at presentation, but this was not addressed in the encounter note, nor was any action or intervention relating to the BP carried out. Diffusion of responsibility included instances in which the specialist visited did not initiate or intensify treatment, explicitly displacing responsibility to a hypertension-managing clinician (ie, primary care physician or cardiology), excluding cases where an urgent referral to the clinician was made. For example, our analysis identified a case where a patient had a visit with a podiatrist and exhibited markedly elevated blood pressure. The podiatrist noted that the patient needed to see their primary care physician, but no follow-up occurred.

#### Patient-Related Scenarios

Patient-related scenarios were defined as instances where clinicians did not initiate or intensify antihypertensive treatment due to considerations related to patient behavior. Under this main content domain, we identified 2 subcategories: patient nonadherence and preference (Figure). Patient nonadherence included instances where the clinician did not intensify intervention due to the patient’s nonadherence to current therapy. For example, our analysis identified a case in which a patient who had previously had adequate BP control while taking metoprolol had not taken his medication in 2 days when he presented with markedly elevated BP and the clinician decided to counsel the patient on adherence rather than modify or intensify treatment at the visit (Table 3). Patient preference included instances where the clinician did not initiate nor intensify intervention due to patient preference.

#### Clinical Complexity–Related Scenarios

These scenarios involve instances where clinicians did not initiate or intensify antihypertensive treatment due to the complexities of the clinical situation. Under this main content domain, we identified 3 subcategories: diagnostic uncertainty with BP measurement, maintenance of current BP intervention, and competing medical priorities (Figure). Diagnostic uncertainty with BP measurement included cases where the clinician did not initiate or intensify treatment due to variation in BP measurements, either at home or in the office. It also reflected situations where clinic BP measurements contradicted home measurements, thus creating uncertainty in determining the true hypertensive status of the patient. Maintenance of current BP intervention included cases where the clinician chose to delay intensifying treatment to observe if current antihypertensives and/or lifestyle modifications would result in BP control. Competing medical priorities included cases in which the clinician chose to delay intensifying treatment due to several competing medical conditions (Table 3). For example, our analysis identified a case where a patient had substantial kidney injury, and the physician decided not to treat the patient’s hypertension due to the kidney condition

## Discussion

This qualitative study provides novel insights into the factors contributing to suboptimal adherence to guidelines among clinicians treating patients with markedly elevated BP in ambulatory settings. Our taxonomy, derived from EHR data, not only categorizes these instances but also describes the factors influencing each scenario of suboptimal adherence. Such a pragmatic framework is poised to inform targeted interventions, thus enhancing adherence and patient outcomes.

Our study advances the existing body of literature in several ways. We have previously detailed various mechanisms through which patients experience persistent hypertension, such as the lack of intensification in pharmacological treatment, failure to implement prescribed therapies, and nonresponse to treatment.^11^ Building on this, the current study specifically illuminates the mechanisms behind clinicians’ failure to treat ambulatory patients with severely elevated BP effectively and explores the reasons for these shortcomings. To our knowledge, this is the first study to develop a taxonomy for categorizing instances of suboptimal clinician adherence to guidelines in managing patients with markedly elevated BP using clinical data. Compared with prior work on clinical inertia,^22,23^ a key strength of this study is its foundation in EHR data. EHRs capture a broad spectrum of clinical interactions across diverse patient demographics, enhancing our findings’ practicality and external validity.^7^ Research based on EHR data can inform more effective clinical decisions by evaluating the quality and cost implications of guideline-conformant care for chronic conditions such as hypertension. Furthermore, EHRs assist in pinpointing the issue of suboptimal clinician guideline adherence in the management of substantially elevated BP and serve as a robust framework for integrating potential EHR-based solutions such as decision support tools.

Various influencing factors were hypothesized for each taxonomized scenario of suboptimal clinician adherence to guidelines in managing severe hypertension. These factors, frequently reported in the literature,^5,24,25^ span health organization, health professional, patient, and guideline contexts. In clinician-related scenarios (eg, did not address and diffusion of responsibility) barriers may include unclear institutional roles, insufficient consultation time, excessive workload, and infrastructure limitations (eTable in Supplement 1). Factors such as clinician autonomy, authority, or role misperceptions can also play a part, alongside unclear guidelines. Patient-related barriers, like nonadherence or preference, might arise from clinician reluctance influenced by patient characteristics, clinician beliefs, fear of complications, and patient unawareness or demotivation. Perceptions of guideline inflexibility also contribute to these barriers. Clinical complexity scenarios, including diagnostic uncertainty, maintenance of interventions, and competing priorities, are affected by organizational issues, reliance on clinical experience over guidelines, patient comorbidities, and guideline perceptions restricting clinical judgment and autonomy.

Our findings underscore the necessity of addressing the multidimensional nature of guideline nonadherence. Under the proposed taxonomy, each category and subcategory of nonadherence scenarios is linked to specific factors and targeted interventions. For example, scenarios affected by organizational factors may improve with robust leadership, clear priorities, sufficient staffing, knowledge-sharing forums, streamlined processes, and regular communicative audits with constructive feedback.^5,26,27,28^ Health organizations can further support clinician adherence by integrating evidence-based decision support tools within EHR systems, such as automated alerts, reminders, and advanced patient portals, along with improved collaborative tools for care teams.^29^ Addressing health professional–level factors involves fostering a willingness to embrace new practices, educating about guidelines, and reinforcing personal accountability.^7,30^ For patient-level factors, strategies include raising health awareness, early education, clear communication about the impact of nonadherence, and peer support. Concerning the guidelines themselves, simplifying their presentation, tailoring them to the local context, and involving end-users in their development can enhance their usability and adherence.^7,31^

Reflecting on the broader implications, this study’s findings can stimulate health care policies aimed at systematizing adherence to guidelines and, thus, improve the quality of care delivered. This is particularly pertinent in light of our identification of implicit bias and structural racism as underlying factors contributing to nonadherence, which are critical to address in the pursuit of equitable health care.^32,33^

### Limitations

While our study’s EHR-based nature substantially enhances its applicability, there are several limitations. First, encounter notes within the EHR may not always provide sufficient detail to conclusively ascertain the intentions or rationale underpinning specific clinical decisions. We did not have information on the characteristics of the physicians, which may play an important role in physician behavior. Additionally, the study’s reliance on the reviewers’ judgment, coupled with the breadth and quality of the referenced meta-review,^6^ could potentially influence the determination of factors contributing to the identified scenarios of nonadherence. The study was conducted at a single academic site, which may limit the applicability of the findings across different types of health care settings. Additionally, our sample predominantly consisted of White patients who were mostly insured. This demographic limitation restricts the generalizability of our findings to more diverse populations.

## Conclusions

In conclusion, by highlighting the multifaceted reasons for suboptimal guideline adherence, our qualitative study provides a foundation for developing nuanced interventions. As we look toward a future of health care that is both evidence-based and patient-centered, it is imperative that we consider the complex interplay of factors at the organizational, professional, patient, and guideline levels that influence clinician behaviors.

## Notes

Supplement 1. **eFigure.** Flow Diagram for Study Population Selection **eTable.** Plausible Influencing Factors for Scenarios of Suboptimal Clinician Guideline Medication Adherence, Based on Analysis of Metareview Findings

Supplement 2.
Data Sharing Statement
