# Supplementary material for: Barriers to Optimal Clinician Guideline Adherence in Management of Markedly Elevated Blood Pressure: A Qualitative Study
Source: JAMA Netw Open. 2024 Aug 6;7(8):e2426135. doi: 10.1001/jamanetworkopen.2024.26135 (PMC11304113; doi:10.1001/jamanetworkopen.2024.26135)
Supplement: Supplement 1. — eFigure. Flow Diagram for Study Population Selection eTable. Plausible Influencing Factors for Scenarios of Suboptimal Clinician Guideline Medication Adherence, Based on Analysis of Metareview Findings [file jamanetwopen-e2426135-s001.pdf]

Supplemental Online Content

Lu Y, Arowojolu O, Qiu X, Liu Y, Curry L, Krumholz HM. Barriers to optimal clinician guideline adherence in management of markedly elevated blood pressure: a qualitative content analysis of electronic health records. *JAMA Netw Open*.2024;7(8):e2426135. doi:10.1001/jamanetworkopen.2024.26135

eFigure. Flow Diagram for Study Population Selection

eTable. Plausible Influencing Factors for Scenarios of Suboptimal Clinician Guideline Medication Adherence, Based on Analysis of Metareview Findings

This supplemental material has been provided by the authors to give readers additional information about their work.

© 2024 Lu Y et al. *JAMA Network Open.*

eFigure. Flow Diagram for Study Population Selection

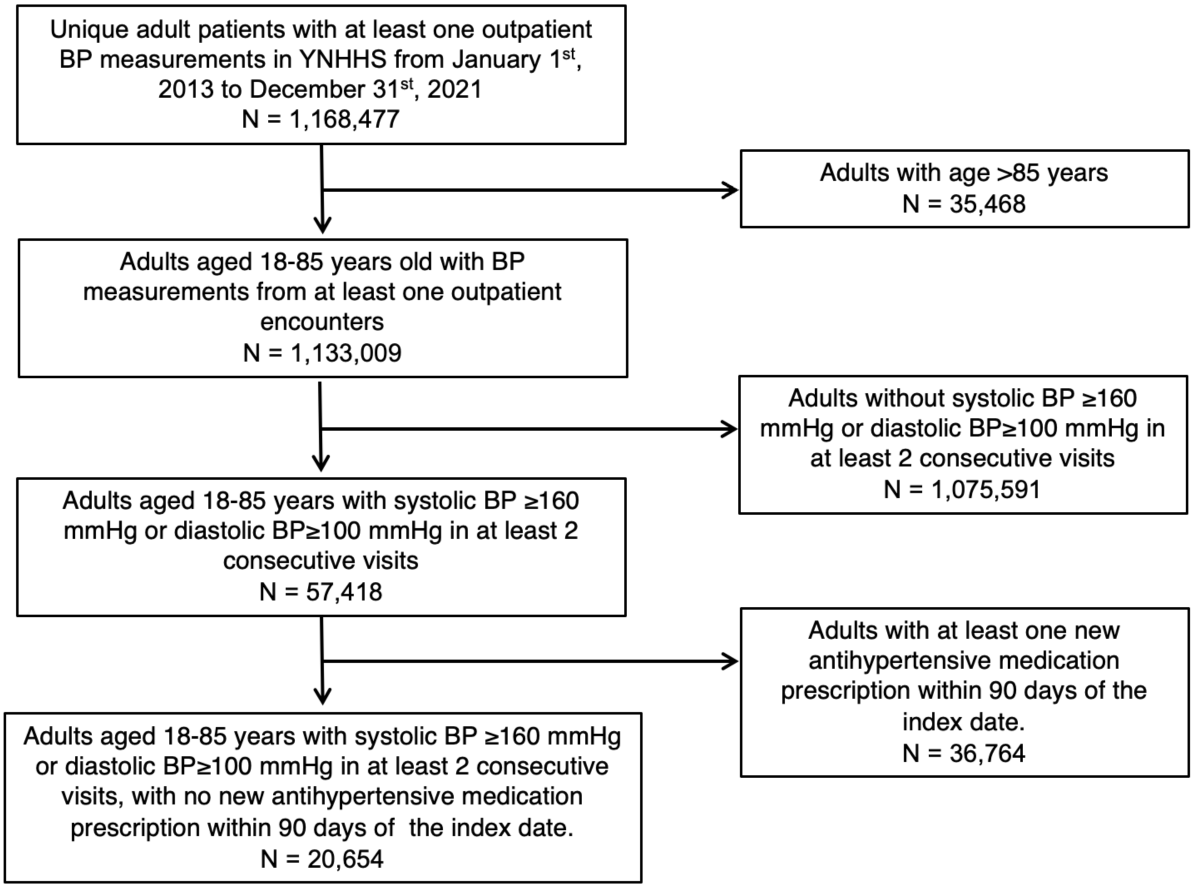

The index date was defined as the date of the 2nd severely elevated BP reading.

© 2024 Lu Y et al. *JAMA Network Open.*

eTable. Plausible Influencing Factors for Scenarios of Suboptimal Clinician Guideline Medication Adherence, Based on Analysis of Metareview Findings

Scenario	Plausible influencing factors*
Did not address	**Health organization context**<br>• Absence of a leader that establishes priorities<br>• Lack of protocols and processes that clearly define the roles within the institution to implement guidelines<br>• Too little time in the medical consultations<br>• Excessive workload<br>• Deficiency in staff continuous education<br>• Deficiencies in the referral of patients to services<br>• Lack of skill and specialist knowledge within services<br>• High turnover of staff that prevents a continuous training process<br>• Lack of coordination and disagreement among staff<br>• Financial constrains for the adoption of new interventions<br>• Lack of availability of interpreters in services<br>• Lack of access to information, lack of mechanisms and systems to support storing of information<br>**Health professional context**<br>• Lack of effective communication, research, and self-learning skills<br>• Little familiarity with guideline recommendations<br>• Lack of autonomy and authority<br>• Belief that intervention was not part of their role<br>**Guideline context**<br>• Lack of clarity of guidelines
Diffusion of responsibility	**Health organization context**<br>• Lack of protocols and processes that clearly define the roles within the institution to implement guidelines<br>• Too little time in the medical consultations<br>• Excessive workload<br>• Deficiency in staff continuous education<br>• Deficiencies in the referral of patients to services<br>• Lack of skill and specialist knowledge within services<br>• Insufficient support from institutions<br>• High turnover of staff that prevents a continuous training process<br>• Limitations of infrastructure

© 2024 Lu Y et al. *JAMA Network Open.*

	• Lack of coordination and disagreement among staff<br>• Lack of availability of interpreters in services<br>• Lack of access to information, lack of mechanisms and systems to support storing of information<br>**Health professional context**<br>• Lack of effective communication, research, and self-learning skills<br>• Little familiarity with guideline recommendations<br>• Lack of autonomy and authority<br>• Belief that intervention was not part of their role<br>**Guideline context**<br>• Lack of clarity of guidelines
Patient non-adherence<br>&<br>Patient Preference	**Health professional context**<br>• Physician's reluctance to use guidelines because of patient factors, self-belief, or fear of complications<br>**Patient context**<br>• Lack of motivation, compliance, and knowledge to follow the recommendations<br>• Unawareness about the health organization characteristics and their disease<br>• Patients' financial situation and occupational status<br>• Depression, anxiety, and fear<br>**Guideline context**<br>• Beliefs that guidelines are too rigid, may not always be practical and cannot be applied on a day-to-day
Diagnostic uncertainty with BP measurement	**Health organization context**<br>• Lack of access to information, lack of mechanisms and systems to support storing of information<br>**Guideline context**<br>• Beliefs that guidelines are too rigid, may not always be practical and cannot be applied on a day-to-day<br>• Guidelines restrict clinical judgment and challenge professional autonomy and limits treatment options
Maintenance of current BP intervention	**Health professional context**<br>• Greater confidence in clinical experience than in guidelines recommendations<br>• Physician's reluctance to use guidelines because of patient factors, self-belief, or fear of complications<br>**Patient context**<br>• Patient comorbidities, mobility problems, polypharmacy, and self-efficacy<br>**Guideline context**

© 2024 Lu Y et al. *JAMA Network Open.*

	• Lack of awareness of the existence of guidelines and clarity of guidelines<br>• Guidelines restrict clinical judgment and challenge professional autonomy and limits treatment options
Competing medical priorities	**Health organization context**<br>• Absence of a leader that establishes priorities<br>**Patient context**<br>• Patient comorbidities, mobility problems, polypharmacy, and self-efficacy

*Synthesized from Correa VC, Lugo-Agudelo LH, Aguirre-Acevedo DC, et al. Individual, health system, and contextual barriers and facilitators for the implementation of clinical practice guidelines: a systematic metareview. *Health Res Policy Syst*. Jun 29 2020;18(1):74. doi:10.1186/s12961-020-00588-8

© 2024 Lu Y et al. *JAMA Network Open.*
