# Supplementary material for: Barriers to Optimal Clinician Guideline Adherence in Management of Markedly Elevated Blood Pressure: A Qualitative Study
Source: JAMA Netw Open. 2024 Aug 6;7(8):e2426135. doi: 10.1001/jamanetworkopen.2024.26135 (PMC11304113; doi:10.1001/jamanetworkopen.2024.26135)
Supplement: Supplement 2. — Data Sharing Statement [file jamanetwopen-e2426135-s002.pdf]

Data Sharing Statement

Lu. Barriers to Optimal Clinician Guideline Adherence in Management of Markedly Elevated Blood Pressure. *JAMA Netw Open*. Published August 06, 2024. doi:10.1001/jamanetworkopen.2024.26135

Data
Data available: No

Additional Information
Explanation for why data not available: Clinical notes of individual patients in electronic health records contain patient identifiable information and cannot be fully deidentified.
